# Tackling control risk problems in non-inferiority trials

**DOI:** 10.1136/bmjmed-2023-000845

**Published:** 2025-06-15

**Authors:** Ian R White, Matteo Quartagno, Abdel G Babiker, Rebecca M Turner, Mahesh KB Parmar, A Sarah Walker

**Affiliations:** 1 MRC Clinical Trials Unit at UCL, London, UK; 2 Nuffield Department of Clinical Medicine, Oxford, UK

**Keywords:** Research design, Statistics, Clinical trial

## Abstract

Non-inferiority trials aim to show that major disease related outcomes with a new intervention are not importantly worse than with standard care. These trials are useful when the new intervention has some advantages over standard care (eg, toxicity, convenience, or cost). The ability to show non-inferiority, however, is sensitive to the control risk, the outcome frequency under standard care. Two control risk problems are described that can make non-inferiority trials underpowered or uninterpretable, and two ways of tackling these problems are outlined. Firstly, the choice of effect measure used to express the non-inferiority margin is critical: the effect measure must be based on understanding both the clinical setting and the implications for sample size. Which effect measures can lead to smaller or larger sample sizes is shown. Secondly, investigators need to consider, and potentially plan for, the possibility that the observed control risk might differ from the anticipated risk at the design stage of the trial. How the non-inferiority margin can be adapted in the trial analysis in a statistically principled manner is shown.

**Key messages**

- Investigators in non-inferiority trials should carefully consider the choice of the effect measure used to define the non-inferiority margin (eg, risk difference or risk ratio)
- For unfavourable binary outcomes (eg, treatment failure), defining a non-inferiority margin with the risk difference rather than the risk ratio gives larger power for the same sample size and the same anticipated differences between randomised arms
- For time-to-event outcomes, using the difference in restricted mean survival time usually gives larger power than using the hazard ratio
- Investigators planning a trial should consider whether the non-inferiority margin or sample size, or both, should be adapted if the observed control risk differs markedly from that anticipated at the design stage, and how this adaptation can be done while retaining the integrity of the trial

## Introduction

 Non-inferiority randomised controlled trials can have two objectives.^1^ The objective considered here is to evaluate whether a new intervention should replace part of an established standard of care because it has advantages from a risk-benefit perspective (eg, lower toxicity, greater patient acceptability, or lower cost). These trials aim to show that major disease related outcomes are not unreasonably compromised with the new intervention. A different objective of non-inferiority trials, important in a regulatory context but not considered here, is to show that a new intervention gives better disease outcomes than placebo, without directly comparing it with placebo.^2^ Non-inferiority trials contrast with superiority trials, which aim to show directly that the new intervention gives better disease outcomes. Non-inferiority trials are widely used^3^ and are especially important where standard care is clinically effective but burdensome on the patient (eg, in tuberculosis^4^) or where diversity in treatment options has public health advantages (eg, in antibiotic prescribing).^2^

Non-inferiority trials have several challenges not encountered in superiority trials.^5 6^ At their design stage, investigators must choose a non-inferiority margin, which is the smallest loss of clinical benefit that is considered unacceptable in view of the other potential advantages of the new intervention. The conduct of a non-inferiority trial must achieve and show good adherence to the control and intervention treatments because poor adherence can dilute the differences between the treatment received in the two arms and therefore tends to bias results towards non-inferiority. For the same reason, the analysis must consider how to deal with non-adherence.^7^

In this article, we show how to tackle two lesser known difficulties in non-inferiority trials, with a particular focus on binary (yes/no) outcomes. These difficulties arise from uncertainty about the outcome risk in the control group, which we call the control risk. We describe each problem before outlining its solution.

## Problem 1: effect measure matters in non-inferiority trials

The Oral versus Intravenous Antibiotics for Bone and Joint Infection (OVIVA) trial was a multicentre randomised controlled trial of antibiotic treatment of bone and joint infections.^8^ The control treatment was intravenous antibiotics, and a 5% control risk was anticipated. The experimental intervention was oral antibiotics, which were believed to be almost as efficacious as intravenous antibiotics. Because of the advantages of the oral route, in particular earlier discharge from hospital, an increase in treatment failures to <10% was considered acceptable. The analysis focused on the risk difference, so the non-inferiority margin was fixed at five percentage points (10% minus 5%). Also, assuming the same true risk in both arms and no loss to follow-up, a non-inferiority trial with 90% power and one sided alpha of 2.5% requires a sample size of 400 participants for each arm (table 1).

**Table 1:** Design and data for non-inferiority trial in antibiotic prescribing

	Experimental intervention	Control intervention
Expected treatment failures (%)	5	5
Borderline acceptable treatment failures (%)	10	5
No of participants randomised	400	400
No (%) hypothetical observed treatment failures	24 (6)	20 (5)

If recruitment to the trial is successful and 24 failures with the experimental intervention and 20 failures with the control intervention are found (these are hypothetical numbers, not the OVIVA results), the estimated risk difference is +1% (95% confidence interval (CI) −2.2% to +4.2%), clearly well below the non-inferiority margin of +5%, and giving a P value of 0.007 for convincing evidence of non-inferiority (table 2). The risk difference, however, is not the only treatment effect measure.^9^ Another view is that the non-inferiority margin is a doubling of risk from 5% to 10%, representing a risk ratio of 2. The estimated risk ratio is 1.20 (95% CI 0.67 to 2.14). This CI includes the non-inferiority margin of 2 and gives a P value of 0.041 for inconclusive evidence of non-inferiority compared with a one sided alpha of 0.025. How can such different conclusions arise from identical data?

**Table 2:** Analysis of hypothetical data from non-inferiority trial in antibiotic prescribing

Effect measure	Non-inferiority margin	Observed effect (95% CI)	Test of non-inferiority (one sided *v* P=0.025)	Evidence of non-inferiority
Risk difference	10%–5%=5%	1.0% (−2.2% to +4.2%)	P=0.007	Convincing
Risk ratio	10%/5%=2	1.20 (0.67 to 2.14)	P=0.041	Not convincing

CI, confidence interval.

The discrepancy arises because a risk difference of 5% and a risk ratio of 2 are only simultaneously true if the control risk is exactly 5%. For example, the data in table 1 are consistent with the control risk being only 3% and the experimental risk being 7%: these values fall within the non-inferiority margin of a risk difference of 5%, but outside the non-inferiority margin of a risk ratio of 2.

## Solution 1: prespecify the effect measure

Problem 1 should be avoided by prespecifying the effect measure in the trial protocol, which is sometimes done implicitly (eg, “The non-inferiority margin is a five percentage points increase from 5% to 10%” or “The non-inferiority margin is a doubling from 5% to 10%”). We recommend stating the intended effect measure explicitly. Stating the effect measure is part of defining the estimand, together with stating the population, treatments being compared, outcome measure (including time point), and handling of intercurrent events.^10^

How should the effect measure be chosen? Clinical considerations are important, and can be understood by varying the control risk slightly. For example, investigators could ask themselves the question: if the control risk is 3%, would the unacceptable experimental arm risk be 8% (a risk difference of 5%) or 6% (a risk ratio of 2)?

Sample size considerations are also important. The extra stringency of the risk ratio has important consequences for the size of the trial. Redesigning the trial in table 1 with 90% power to exclude a relative increase of 2 in the risk would require a sample size of 832 (rather than 400) for each arm with the same assumptions (table 3). Sample size based on the risk ratio is in general larger than the sample size based on the risk difference for unfavourable outcomes. For favourable outcomes, sample size is instead smaller when based on the risk ratio (better termed the success ratio) than on the risk difference.^11^ If clinical considerations do not dictate the choice of effect measure, then a choice resulting in smaller sample size is reasonable. The online supplemental appendix gives the code for doing these sample size calculations.

**Table 3:** Total sample sizes required for non-inferiority trial in antibiotic prescribing

Control risk (%)	Unacceptable experimental risk (%)	Based on risk difference		Based on risk ratio	
		Non-inferiority margin (%)	Total sample size	Non-inferiority margin	Total sample size
**Unfavourable outcome**					
5	≥10	5	800	2	1664
15	≥20	5	2144	1.33	2878
25	≥30	5	3154	1.2	3794
**Favourable outcome**					
10	≤5	−5	1514	0.5*	788
20	≤15	−5	2690	0.75*	2032
30	≤25	−5	3532	0.83*	2952

* The risk ratio for a favourable outcome is better termed the success ratio.

## Problem 2: observed control risk might not be anticipated

Despite investigators’ best efforts to anticipate the control risk correctly, sometimes its observed value differs importantly from what was anticipated. At the interim analysis, this finding might be noted by an independent data monitoring committee with access to unblinded data or by the investigators with the pooled data and assuming no treatment effect. At the final analysis, the differing control risk will become clear.

**Figure 1:**
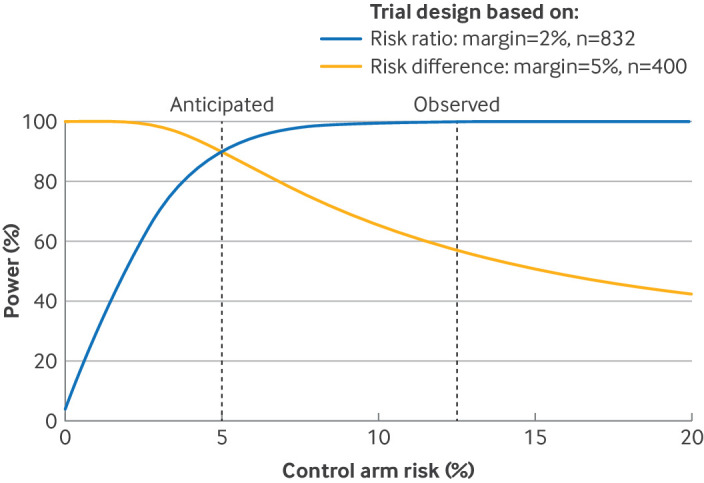
Power calculation for two non-inferiority trials with unfavourable outcome as the control risk varies: trial designed with the risk difference with a non-inferiority margin of 5% and trial designed with the risk ratio with a non-inferiority margin of 2. n=sample size for each arm

This difficulty is particularly likely if a trial is being designed in a discipline that has few previous trials, or the inclusion criteria differ materially from previous trials. For OVIVA, no other trials could inform the control risk, but a pilot in the main centre, which had substantial management and treatment experience, suggested that a 5% control risk was reasonable. The interim analysis, however, including many centres with less experience, found a larger overall risk (and hence by assumption control risk) of 12.5%. Now a non-inferiority margin of 5% as a risk difference means that the experimental risk must be <17.5%, which clinicians might consider too stringent given the other potential benefits of the intervention. On the other hand, a non-inferiority margin of 2 as a risk ratio means that the experimental risk must be <25%, which clinicians might consider too lax because it corresponds to one in four patients failing, rather than one in eight. Also, the power of the trial to show non-inferiority with the risk difference is reduced from the anticipated 90% to <60% (figure 1). On the other hand, the power of a larger trial designed with the risk ratio would be increased from the anticipated 90% to almost 100%.

Hence the control risk being higher than anticipated means low power to detect a possibly too small non-inferiority margin if the risk difference is used, or high power to detect a possibly too large non-inferiority margin if the risk ratio is used. The opposite problems occur if the control risk is lower than anticipated. We now show how to adapt the non-inferiority margin to avoid these problems, which are only identified after a trial has started, while retaining the statistical integrity of the trial.

## Solution 2: respond to unanticipated control risk

What can investigators do if an interim or final analysis shows that the control risk observed so far in the trial differs substantially from the anticipated risk when the trial was designed? If the original non-inferiority margin remains clinically relevant given the observed control risk, but power is now substantially lower than designed, then the investigators have few options: at an interim analysis, the investigators might consider stopping the trial for futility or seek funding to increase the size of the trial, or otherwise, at the final analysis, simply accept the loss of power.

In some cases, however, the fact that the control risk is different to that anticipated allows reconsideration of the original non-inferiority margin. In the OVIVA trial,^8^ with the interim analysis showing a control risk of about 12.5% rather than the anticipated 5%, the investigators chose to adapt the risk difference non-inferiority margin from 5% to 7.5%, based on clinical trade-offs for acceptable failure rates given the other advantages of oral treatment.^9^ The data monitoring committee, the trial steering committee, and the research ethics committee explicitly agreed to this change.^12^

Adapting the non-inferiority margin taking into consideration the estimated treatment effect is inappropriate; adapting the non-inferiority margin considering the control risk or overall risk alone is appropriate, but could lead to an inflated type 1 error.^9^ We recommend that investigators prespecify a procedure for adapting the non-inferiority margin, stating when the decision would be taken, by whom, and on what evidence; what size of discrepancy would trigger a change in the non-inferiority margin; how the non-inferiority margin would be changed; and how the type 1 error rate would be controlled.^11^ Before suggesting a procedure, we introduce a useful visual aid.

### Visual aid: non-inferiority frontier graph

**Figure 2:**
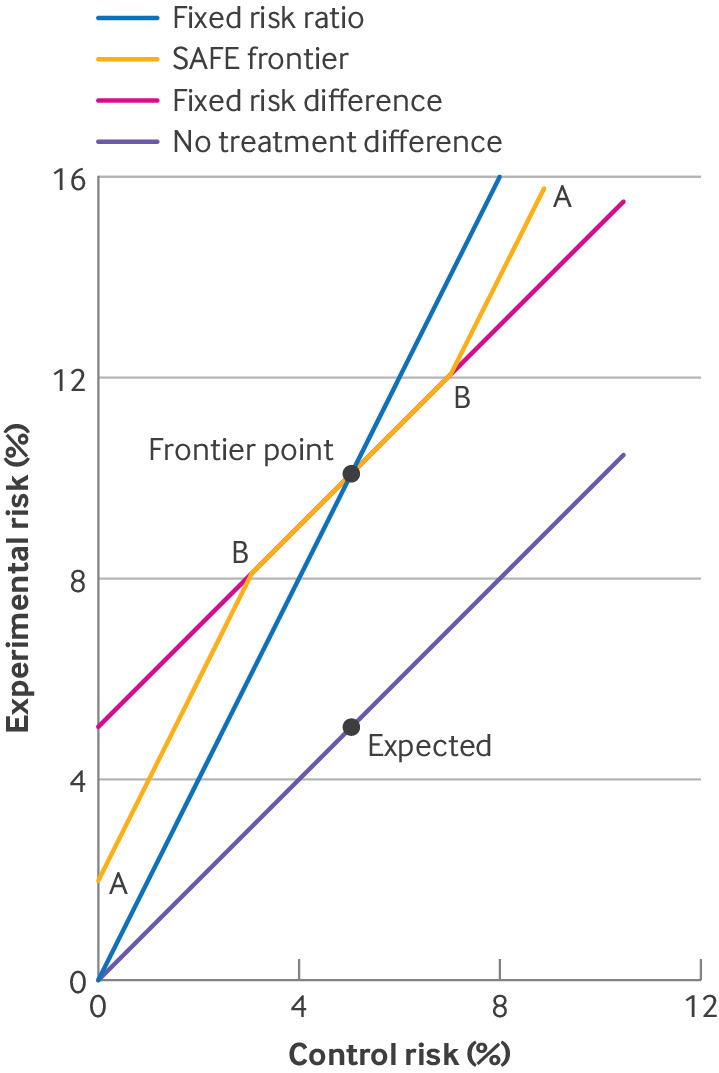
Three possible non-inferiority frontiers: fixed risk difference frontier, fixed risk ratio frontier, and smooth away from expected (SAFE) frontier (marked A). The SAFE frontier follows the fixed risk difference frontier around the expected control risk, because clinical interpretation of the non-inferiority margin is unchanged in this region, and then bends away smoothly at points B to pass the frontier points A

In figure 2, we show the experimental risk and control risk in a two way graph.^11^ Each point on the graph represents a potential truth. The line of points representing no difference between the experimental and control treatments (no treatment difference) includes the expected point where both risks are 5%. When the control risk is 5%, non-inferiority is defined by an experimental risk of <10%: we call 10% the frontier point. Another line of points represents a risk difference equal to the non-inferiority margin of 5% (fixed risk difference). A steeper line of points represents a risk ratio equal to the non-inferiority margin of 2 (fixed risk ratio). Points below either line indicate non-inferiority with that effect measure. Both lines pass through the frontier point but they differ elsewhere.

The non-inferiority frontier graph explains problem 1: for control risk values of <5%, the expected point is nearer to the fixed risk ratio frontier than to the fixed risk difference frontier, making it harder to reject the former. The graph also suggests a solution to problem 2: we can use an intermediate frontier between the fixed risk ratio and fixed risk difference frontiers.

### Proposal: smooth away from expected (SAFE) frontier

The trial team should determine at the design stage of the study, on clinical grounds, what non-inferiority means at different control risks. This decision should be broadly discussed by clinicians, patients, and statisticians. Based on the other potential advantages of the new intervention, the investigators might decide that if the control risk is 1% then non-inferiority requires the experimental risk to be <4%, and if the control risk is 9% then non-inferiority requires the experimental risk to be <16%. The trial team can add these as two more frontier points (marked A in figure 2). Figure 2 shows an example of the new SAFE frontier.^13^ The SAFE frontier follows the fixed risk difference frontier around the expected control risk, because clinical interpretation of the non-inferiority margin is unchanged in this region, and then bends away smoothly at points B in figure 2 (here drawn where control risk is the expected value ±~2%) to pass the frontier points A. Choice of points A and B should reflect clinical understanding, but placing points B close to the frontier point inflates sample size.

Table 4 summarises the SAFE frontier and analysis.^13^ Here, to control type 1 errors, we choose a smaller nominal significance level (0.75%) if the non-inferiority margin is adapted, and otherwise a standard 2.5% nominal significance level. Adapting the non-inferiority margin is increasingly likely as the control arm risk moves away from the expected 5%. Power is slightly reduced (from 90% to 87%) if the control risk is 5%, but does not fall to <70% for any control risk. Type 1 error is controlled at <5%, but not at <2.5%. Table 5 summarises steps that can be taken to tackle the control risk problems, and box 1 suggests wording for a trial protocol with a SAFE frontier.

**Table 4:** Performance of the smooth away from expected (SAFE) frontier shown in figure 2

	Control arm risk (%)									
	1	2	3	4	5*	6	7	8	9	10
Non-inferiority margin (%)	3	4	4.94	5	5	5	5.06	6	7	8
Probability of adapting margin (%)	100	94	58	19	7	18	45	74	91	98
Power achieved (%)	74	78	82	87	87	82	75	71	74	80
Type 1 error achieved (%)	1.9	3.1	2.3	2.3	2.2	2.6	4.4	4.6	4.6	4.7

* Expected control arm risk.

**Table 5:** Steps to tackle control risk problems

Trial stage	Action
Design	Choose an effect measure and non-inferiority margin that are clinically reasonable and statistically efficient. Specify these quantities clearly in the trial protocol
	Based on the strength of evidence supporting the choice of control risk, consider, and ideally plan, whether and how the non-inferiority margin will be adapted if the observed control risk differs from that anticipated. Specify this approach clearly in the trial protocol, including if no adaptation is planned
Interim analysis	The independent data monitoring committee should compare the observed control risk with that anticipated. If the difference is substantial, recommending the following actions should be considered
	If non-inferiority margin is still appropriate and power will be lower than anticipated: Seek funding to increase the size of the trial Stop the trial for futility
	If non-inferiority margin is still appropriate and power will be higher than anticipated: Consider doing the final analysis earlier
	If non-inferiority margin is not appropriate: Change the non-inferiority margin taking into consideration decisions made about prespecifying adaptations
Final analysis	Compare the observed control risk with the anticipated control risk. If the difference is substantial, consider the following actions
	If non-inferiority margin is still appropriate and power will be lower than anticipated: Accept lost power
	If non-inferiority margin is still appropriate and power will be higher than anticipated: No action required
	If non-inferiority margin is not appropriate: Reconsider the non-inferiority margin, consulting with trial committees if no prespecification of adaptations

Box 1 **Suggested wording for a trial protocol alongside a graph of the smooth away from expected (SAFE) frontier**

Analysis will use the risk difference. The anticipated control risk is 5% and the non-inferiority margin is five percentage points. If the observed control risk differs from that anticipated in the sample size calculation by more than ~2 percentage points, then we will adapt the non-inferiority margin according to the SAFE frontier shown in the graph. In this case, we will control the type 1 error rate by using a smaller nominal significance level, and report results with the adapted non-inferiority margin.^13^

## Discussion

Prespecifying the effect measure in a non-inferiority trial is important. We have focused on the risk difference and risk ratio. Another effect measure for binary data is the odds ratio. The odds ratio gives similar results to the risk ratio for all examples in this paper, because the odds ratio and risk ratio perform similarly for low frequency outcomes.^14^ In general, a non-inferiority margin expressed as an odds ratio is difficult to interpret,^15^ and the risk difference or risk ratio should be preferred in non-inferiority trials. Another possibility is the averted infections ratio.^16^ Recasting the trial as superiority with a composite outcome is attractive but problematic.^17^

We assumed an analysis which simply compares risks. Power might be gained by adjusting for baseline covariates. Covariate adjustment is usually done in a logistic regression analysis that outputs conditional odds ratios: the results should be converted to the chosen effect measure (eg, risk difference or risk ratio) with standardisation.^18^

We assumed a binary outcome. Time-to-event outcomes raise similar problems: these outcomes are often analysed with the hazard ratio, which behaves like the risk ratio. Expressing the non-inferiority margin based on the difference in restricted mean survival time has been shown to reduce sample size requirements, sometimes substantially.^19^ Quantitative outcomes also require a decision on whether non-inferiority is to be defined by a difference or ratio of means.

Adapting the non-inferiority margin during the trial could be problematic. A particular concern is that investigators will choose a non-inferiority margin that gives the desired results, which is clearly unacceptable. To make an adapted non-inferiority margin acceptable to stakeholders, the adaptation procedure needs to be free from bias, transparent, and prespecified. Adaptation might complicate the reporting of a non-inferiority trial.

We described a non-inferiority margin adaptation procedure based on the SAFE frontier and overall blinded data. Non-inferiority margin adaptation alone can lead to type 1 error inflation, but special analysis methods can control the type 1 error rate.^13^ Sample size re-estimation or doing the final analysis earlier could have unexplored implications for type 1 errors.

Our arguments are specific to non-inferiority trials demonstrating advantages from a risk-benefit perspective. In regulatory trials, where the aim is an indirect comparison with placebo, the effect measure is typically the same as in trials of control versus placebo, and large differences in control risk are likely to question the credibility of the trial.

### Conclusions

Non-inferiority trials have greater risks to validity than superiority trials, but the design of the trial can reduce these risks. Careful choice of effect measure can avoid unnecessarily large sample size requirements. Advance consideration of what will be done if the control risk differs from its anticipated value can avoid trials being underpowered or uninterpretable. The SAFE frontier is one way to avoid these problems.

## Supplementary material

10.1136/bmjmed-2023-000845 online supplemental file 1

## Data Availability

Data sharing not applicable as no datasets generated and/or analysed for this study.

## References

[1] Tweed CD, Quartagno M, Clements MN (2024). Exploring different objectives in non-inferiority trials. BMJ.

[2] Department of Health and Human Services: Food and Drug Administration (2016). Non-inferiority clinical trials to establish effectiveness: guidance for industry. https://www.fda.gov/regulatory-information/search-fda-guidance-documents/non-inferiority-clinical-trials.

[3] Rehal S, Morris TP, Fielding K (2016). Non-inferiority trials: are they inferior? A systematic review of reporting in major medical journals. BMJ Open.

[4] Nunn AJ, Meredith SK, Spigelman MK (2008). The ethics of non-inferiority trials. Lancet.

[5] Mauri L, D’Agostino RB (2017). Challenges in the Design and Interpretation of Noninferiority Trials. N Engl J Med.

[6] Macaya F, Ryan N, Salinas P (2017). Challenges in the Design and Interpretation of Noninferiority Trials: Insights From Recent Stent Trials. J Am Coll Cardiol.

[7] Hernán MA, Robins JM (2017). Per-Protocol Analyses of Pragmatic Trials. N Engl J Med.

[8] Li HK, Scarborough M, Zambellas R (2015). Oral versus intravenous antibiotic treatment for bone and joint infections (OVIVA): study protocol for a randomised controlled trial. Trials.

[9] Li Z, Quartagno M, Böhringer S (2022). Choosing and changing the analysis scale in non-inferiority trials with a binary outcome. *Clin Trials*.

[10] Kahan BC, Hindley J, Edwards M (2024). The estimands framework: a primer on the ICH E9(R1) addendum. BMJ.

[11] Quartagno M, Walker AS, Babiker AG (2020). Handling an uncertain control group event risk in non-inferiority trials: non-inferiority frontiers and the power-stabilising transformation. Trials.

[12] Li H-K, Rombach I, Zambellas R (2019). Oral versus Intravenous Antibiotics for Bone and Joint Infection. N Engl J Med.

[13] Quartagno M, Chan M, Turkova A (2023). The Smooth Away From Expected (SAFE) non-inferiority frontier: theory and implementation with an application to the D3 trial. Trials.

[14] Cornfield J (1951). A method of estimating comparative rates from clinical data; applications to cancer of the lung, breast, and cervix. J Natl Cancer Inst.

[15] Sackett D, Deeks J, Altman D (1996). Down with odds ratios!. Evid Based Med.

[16] Dunn DT, Glidden DV, Stirrup OT (2018). The averted infections ratio: a novel measure of effectiveness of experimental HIV pre-exposure prophylaxis agents. Lancet HIV.

[17] Phillips PPJ, Morris TP, Walker AS (2016). DOOR/RADAR: A Gateway Into the Unknown?. Clin Infect Dis.

[18] Morris TP, Walker AS, Williamson EJ (2022). Planning a method for covariate adjustment in individually randomised trials: a practical guide. Trials.

[19] Quartagno M, Morris TP, Gilbert DC (2023). A comparison of different population-level summary measures for randomised trials with time-to-event outcomes, with a focus on non-inferiority trials. *Clin Trials*.

