# Supplementary material for: Tackling control risk problems in non-inferiority trials
Source: BMJ Med. 2025 Jun 15;4(1):e000845. doi: 10.1136/bmjmed-2023-000845 (PMC12314831; doi:10.1136/bmjmed-2023-000845)
Supplement: online supplemental file 1 [file bmjmed-4-1-s001.pdf]

Online appendix: R code for Table 3.

```
# Install dani v1.0-0 from https://github.com/Matteo21Q/dani. It will be on CRAN in the future.

library(dani)

# The commands below assume a 2.5% one-sided significance level, 90% power and 1:1 allocation: these can be changed by the sig.level=, power= and r= options.

# They also assume a favourable outcome. For an unfavourable outcome we would specify the unfavourable=FALSE option and set the NI margin to be negative.

# The first 3 arguments are: expected control risk, expected experimental risk, NI margin using the chosen summary measure.

samplesize.NI.binary(0.05, 0.05, 0.05, summary.measure="RD", test.type="Wald")

samplesize.NI.binary(0.05, 0.05, 2, summary.measure="RR", test.type="Wald")

samplesize.NI.binary(0.15, 0.15, 0.05, summary.measure="RD", test.type="Wald")

samplesize.NI.binary(0.15, 0.15, 4/3, summary.measure="RR", test.type="Wald")

samplesize.NI.binary(0.25, 0.25, 0.05, summary.measure="RD", test.type="Wald")

samplesize.NI.binary(0.25, 0.25, 1.2, summary.measure="RR", test.type="Wald")
```
