# Supplementary material for: Tackling control risk problems in non-inferiority trials
Source: BMJ Med. 2025 Jun 15;4(1):e000845. doi: 10.1136/bmjmed-2023-000845 (PMC12314831; doi:10.1136/bmjmed-2023-000845)
Supplement: Supplementary file 2 [file bmjmed-2023-000845.reviewer_comments.pdf]

**PEER REVIEW HISTORY**

BMJ Medicine publishes all reviews undertaken for accepted manuscripts. Reviewers are asked to complete a checklist review form and are provided with free text boxes to elaborate on their assessment. These free text comments are reproduced below.

**ARTICLE DETAILS**

| | |
|---|---|
| **TITLE (PROVISIONAL)** | Tackling control risk problems in non-inferiority trials |
| **AUTHORS** | White, Ian; Quartagno, Matteo; Babiker, Abdel; Turner, Becky; Parmar, Mahesh; Walker, Ann |

**VERSION 1 - REVIEW**

| | |
|---|---|
| **REVIEWER NAME** | Phillips, Patrick |
| **REVIEWER AFFILIATION** | UCSF |
| **REVIEWER CONFLICT OF INTEREST** | None |
| **AI DISCLOSURE** | No |
| **DATE REVIEW RETURNED** | 08-Feb-2024 |

| | |
|---|---|
| **GENERAL COMMENTS** | The non-inferiority design is pervasive in medicine yet also poorly understood and poorly implemented. It is a necessary burden in many disease areas with somewhat effective standards of care where alternative interventions provide public health benefit that is not easily measurable in a superiority trial.<br><br>This manuscript brings previously published statistical methodology that improves how we handle uncertainty in the control arm risk to a general medical audience and is therefore a welcome addition to the literature. The recommendations are practical, sound, and backed up by careful and methodical statistical methodology.<br><br>The manuscript is, however, overly brief in places and would benefit from a little more discussion.<br><br>Major comments:<br>• Page 3. Line 23-24. The description of the margin as 'the smallest loss of clinical benefit that is considered unacceptable in view of the new intervention's other potential advantages' is commonly accepted and very reasonable. Regulatory agencies, however, take a slightly different view. For example, the FDA guidance on non-inferiority states that the margin 'can be no larger than the presumed entire effect of the active control in the NI study'. The guidance goes into excruciating detail of how determination of the margin must depend on historical evidence of the effect of the active control compared to placebo. This might be considered an overly restrictive perspective of non-inferiority margins, but is nevertheless a common regulatory perspective. How do the authors' recommendations fit into this regulatory perspective? How would the SAFE frontier be interpreted in relation to the active control effect size (as compared to placebo)? To quote from the FDA guidance 'a finding of non-inferiority means that the test drug has an effect greater than 0' - would this interpretation still apply if investigators used the SAFE frontier rather than a fixed margin? Some discussion in relation to a regulatory perspective would be invaluable. |

| | |
|---|---|
| | • The limitations section is very brief and there is no discussion about other methods for handling some of these problems in NI trials from the literature. One obvious method is use of the Averted Infections Ratio (arising from work for the MRC CTU). Other methods that are often posed as solving some of the problems raised by the authors include DOOR/RADAR or Bayesian approaches to NI. The non-statistical and non-trialist reader would benefit from brief discussion of advantages and disadvantages of the recommendations in this manuscript compared to other methods.<br>• The limitations section is too short. The authors should also mention other perceived limitations - even if these are minor. Is there a problem in interpretation by a non-statistical audience, or publication, or adoption into practice guidelines with use of a margin of non-inferiority that is not fixed? Is there a risk of changes in trial conduct or patient management during a trial if the margin of non-inferiority is changed after an interim analysis (a potential problem with any adaptive design)? How would regulatory agencies view a changing margin in a non-inferiority trial?<br>• Page 4. Line 41-42, it is stated that sample size is 'smaller for favorable outcomes', but this seems misleading and thus could give the wrong impression to a non-statistical reader. Table 3 doesn't compare like for like between unfavorable outcome sample sizes and favorable outcome sample sizes. A sample size with 10% control arm risk and 5% margin is the same whether the 10% is favorable or unfavorable outcomes. Please expand on this statement in lines 41-42 to explain how and under what circumstances this is correct.<br><br><br>Minor comments:<br>• The CONSORT guidelines recommend reporting of both absolute and relative effect sizes in a clinical trial report. If an investigator uses the SAFE frontier, would this recommendation still apply?<br>• State somewhere that sample size calculations are done under the assumption that the expected risk in the experimental arm is the same as, and varies with, the expected risk in the control arm.<br>• Suggest moving the OVIVA trial example to the section 'Problem 2' rather than 'Solution 2' as it might help the reader understand the problem better.<br>• Consider adding power for SAFE frontier to Figure 1.<br>• Suggest including a real trial example for 'Problem 1' to help the reader appreciate the problem. |

| | |
|---|---|
| **REVIEWER NAME** | van Geloven, Nan |
| **REVIEWER AFFILIATION** | Leiden University Medical Center |
| **REVIEWER CONFLICT OF INTEREST** | None |
| **AI DISCLOSURE** | No |
| **DATE REVIEW RETURNED** | 29-Mar-2024 |

| | |
|---|---|
| **GENERAL COMMENTS** | I appreciate the opportunity to review this manuscript on the challenge of control risk dependency in non-inferiority trials. I agree with the authors that this is an often overlooked aspect and while the methods presented in the manuscript have been published in statistical literature, it is good that authors wrote this paper targeted at a more broader readership.<br>In general I think the paper addresses the audience well, in the |

sense that statistical points are explained accessibly with clear numerical examples of when NI results differ between different effect measures. I do think at some points too much detail is removed. I indicate those places below. I think they need more attention.

- the solution to ‘problem 1’ is suggested to be solved by prespecifying the scale. I think this is typically done in trial protocols, but the problem comes back in the analysis phase where eg due to the desire to use a regression approach reporting changes to a different effect measure. I think this needs to be discussed too.
- In light of this, regression approaches often lead to odds ratios. Authors do not consider this effect measure. Do they advise against it?
- In problem 2: not always clear when authors consider interim analysis and when final analysis. Eg P1L52 ‘eventually’, does this exclude interim? P3L54: ‘is importantly different’, does that mean at interim at final analysis or in population? P4L24 ‘after the trial has started’. For interim analysis, the issue of whether or not to deblind the results (needed to actually observe the control risk) is not discussed. And in table 4 it is advised at interim analysis to ‘compare the observed control risk with that anticipated’. Without further referencing to issues with deblinding, this could be interpreted as an encouragement to deblind the data at interim analysis. I don’t think that is advisable in general, can authors clarify this?
- Relatedly, there is little attention in the paper to type I error control. Probably specific procedures needed for this when using the SAFE frontier? Can this be added to one of the examples or the figure 2? End of ‘limitations’ section seems to suggest no good method exists for it yet? Can it then be used already in practice, what is the current advise on it? I also wonder if the advice to seek funding to enlarge the trial based on observed control risk during interim (table 4) will have any consequences for type I error control. And the same worry for the advice to bring the final analysis forward in case of power advantage anticipated at interim analysis.
- In the suggested wording in the Box, there is no mentioning of the actual margin, neither of the location of points B. This seems prone to incomplete reporting if researchers would copy this text.
- The end of the paper is very short and only mentions something about the SAFE frontier. Intro of the paper seems to address a more broad problem, not only the SAFE frontier. The closing of the paper could use some more attention and should link to intro.
- The example code in the appendix should include the full write out of the default arguments, eg alpha, one or 2 sided, positive or negative event, etc.

Some further minor suggestions for the paper:

- in some places the issue is described as ‘the control risk problem’, eg title and P6L1. While in the paper ‘2 problems’ are mentioned. This wording is bit confusing. See also P4L24 ‘to avoid these problems’. Both problem 1 and problem 2?
- P2L9 ‘broadening therapeutic toolkit’: vague wording. Also the setting where non-inferiority is used to indirectly prove superiority over placebo is not mentioned/referred to.

-- P4L6: ‘are only the same’. They are never the same, maybe better to reword into ‘will both be true at the same time’ or smth like that?

- P5L36: should determine upfront
- P5L50: may not be clear to all readers what ‘this procedure’ means, it is worthwhile to spell out

-P5L45: it was not clear from the example so far that risk difference

| | |
|---|---|
| | was the originally preferred scale. Also not so clear where to place points B, and why plus or minus 2%?<br>- P5L48: 'typically, we expect one of the frontiers in the Figure would be adequate.' Why is this, there can be many others? I think the point is more that trialists need to be aware that they need to choose a curve, not only a point. They can in principle choose any, but these are 3 simple candidates?<br>- table 4 design: here referred to 'scale' at other places 'effect measure'. I suggest to unify. Also here needs to be clear that the size of NI margin needs to be specified.<br>- in table 4 indicated that it is uncommon that different control risk leads to higher power. Why would than be, is it not a symmetric problem?<br>- Figure 1: legend needs to be clear about that risk ratio is for negative or positive outcome |

| | |
|---|---|
| **REVIEWER NAME** | Riley, Richard |
| **REVIEWER AFFILIATION** | University of Birmingham, Institute of Applied Health Research |
| **REVIEWER CONFLICT OF INTEREST** | None |
| **AI DISCLOSURE** | No |
| **DATE REVIEW RETURNED** | 06-Jun-2024 |

| | |
|---|---|
| **GENERAL COMMENTS** | This is a well-written and important article that I think would be well cited and have an immediate impact. It would fit well as a methodology piece in the journal, as introduces interesting issues/problems with sample size and non-inferiority trials, with potential solutions that are well justified. I see that two reviewers already given pertinent comments, so I only have a few suggestions to add<br><br>1) Add a concluding remarks section, just to summarise the overall article and what has been discussed – otherwise ends rather abruptly<br>2) The word estimand is not used, which I am surprised about. I think the issues should be framed in terms of the estimand for the NI trial, and how this might need to change depending on the particular control risk issue that arises<br>3) Odds ratios are not considered, but many trials focus on the OR as an estimand. How does this impact things?<br>4) Similarly, if the estimand is a conditional effect (e.g. adjusted for baseline age, stage of disease), how might this impact the issues and solutions?<br>5) Could we have a box of recommendations perhaps?<br>6) Worth emphasising that risk by a particular time-point, for both binary or tte outcomes, and so the estimand needs to be defined in terms of risk by timepoint stated?<br>7) The title mentions the word 'resilience', but this is not referred to in the actual paper. It's not clear what is meant by this – so perhaps 'Tackling the control risk in non-inferiority trials' would be better, but this is a minor point (tackle is mentioned 4 times!) |

## VERSION 1 – AUTHOR RESPONSE

**Reviewer: 1**

Patrick Phillips, UCSF

*The non-inferiority design is pervasive in medicine yet also poorly understood and poorly implemented. It is a necessary burden in many disease areas with somewhat effective standards of care where alternative interventions provide public health benefit that is not easily measurable in a superiority trial.*

*This manuscript brings previously published statistical methodology that improves how we handle uncertainty in the control arm risk to a general medical audience and is therefore a welcome addition to the literature. The recommendations are practical, sound, and backed up by careful and methodical statistical methodology.*

*The manuscript is, however, overly brief in places and would benefit from a little more discussion.*

**Major comments:**

1. *Page 3. Line 23-24. The description of the margin as 'the smallest loss of clinical benefit that is considered unacceptable in view of the new intervention's other potential advantages' is commonly accepted and very reasonable. Regulatory agencies, however, take a slightly different view. For example, the FDA guidance on non-inferiority states that the margin 'can be no larger than the presumed entire effect of the active control in the NI study'. The guidance goes into excruciating detail of how determination of the margin must depend on historical evidence of the effect of the active control compared to placebo. This might be considered an overly restrictive perspective of non-inferiority margins, but is nevertheless a common regulatory perspective. How do the authors' recommendations fit into this regulatory perspective? How would the SAFE frontier be interpreted in relation to the active control effect size (as compared to placebo)? To quote from the FDA guidance 'a finding of non-inferiority means that the test drug has an effect greater than 0' - would this interpretation still apply if investigators used the SAFE frontier rather than a fixed margin? Some discussion in relation to a regulatory perspective would be invaluable.*

We agree that this second view of a NI trial was omitted. Our arguments are unlikely to apply to this regulatory view, since the effect measure is likely to need to be the same as in the historical evidence about the active control vs placebo. We have clarified this in the introduction by adding:

> Non-inferiority randomised controlled trials can have two objectives(1). The objective considered here is to evaluate whether a new intervention should replace part of an established standard of care, because it has advantages from a risk-benefit perspective: for example, lower toxicity, greater patient acceptability, or lower cost. Such trials aim to show that major disease-related outcomes are not unreasonably compromised with the new intervention. A different objective of non-inferiority trials, important in a regulatory context but not considered here, is to demonstrate that a new intervention gives better disease outcomes than placebo without directly comparing it with placebo(2).

and we've revisited the regulatory view in the discussion by adding:

> Our arguments are specific to NI trials demonstrating advantages from a risk-benefit perspective. In regulatory trials whose aim is an indirect comparison with placebo, the effect measure is typically the same as in trials on control versus placebo, and large differences in control risk are likely to cast doubt on the trial's credibility.

2. *The limitations section is very brief and there is no discussion about other methods for handling some of these problems in NI trials from the literature. One obvious method is use of the Averted Infections Ratio (arising from work for the MRC CTU). Other methods that are often posed as solving some of the problems raised by the authors include DOOR/RADAR or Bayesian approaches to NI. The non-statistical and non-trialist reader would benefit from brief discussion of advantages and disadvantages of the recommendations in this manuscript compared to other methods.*

We have changed the Limitations section to a more detailed Discussion section (pasted in response to 3). This covers the AIR and DOOR/RADAR ideas.

3. *The limitations section is too short. The authors should also mention other perceived limitations - even if these are minor. Is there a problem in interpretation by a non-statistical audience, or publication, or adoption into practice guidelines with use of a margin of non-inferiority that is not fixed? Is there a risk of changes in trial conduct or patient management during a trial if the margin of non-inferiority is changed after an interim analysis (a potential problem with any adaptive design)? How would regulatory agencies view a changing margin in a non-inferiority trial?*

As noted, we have changed the Limitations section to a more detailed Discussion section. This covers difficulties in reporting, interpretation and credibility:

> Pre-specifying the effect measure in a NI trial is important. We have focussed on the risk difference and risk ratio. Another effect measure for binary data is the odds ratio. This gives very similar results to the risk ratio for all examples in this paper, since the odds ratio and risk ratio perform very similarly for low-frequency outcomes[14]. In general, a non-inferiority margin expressed as an odds ratio is hard to interpret[15], and the risk difference or risk ratio should be preferred in non-inferiority trials. Another possibility is the averted infections ratio[16]. Recasting the trial as superiority using a composite outcome is attractive but problematic[17].
>
> We assumed an analysis which simply compares risks. Power may be gained by adjusting for baseline covariates. This is usually done in a logistic regression analysis, which outputs conditional odds ratios: the results should be converted to the chosen effect measure (e.g. risk difference or risk ratio) using standardisation[18].
>
> We assumed a binary outcome. Time-to-event outcomes raise similar issues: they are often analysed using the hazard ratio, which behaves like the risk ratio. Expressing the non-inferiority margin using the difference in restricted mean survival time has been shown to reduce sample size requirements, sometimes substantially[19]. Quantitative outcomes also require a decision whether non-inferiority is to be defined using a difference or ratio of means.
>
> Modifying the non-inferiority margin during the trial could be problematic. A particular concern is that investigators will choose a non-inferiority margin that gives the desired results, which is clearly unacceptable. To make a modified non-inferiority margin acceptable to stakeholders, the modification procedure needs to be free from bias, transparent and pre-specified. This may complicate the reporting of a non-inferiority trial.
>
> We described a non-inferiority margin modification procedure based on the SAFE frontier and overall blinded data. Non-inferiority margin modification alone can lead to type 1 error inflation, but special analysis methods can control the type 1 error rate[13]. Sample size re-estimation or bringing final analysis time forward could have unexplored implications for type 1 error.
>
> Our arguments are specific to NI trials demonstrating advantages from a risk-benefit perspective. In regulatory trials whose aim is an indirect comparison with placebo, the effect measure is typically the same as in trials on control versus placebo, and large differences in control risk are likely to cast doubt on the trial's credibility.

4. *Page 4. Line 41-42, it is stated that sample size is 'smaller for favorable outcomes', but this seems misleading and thus could give the wrong impression to a non-statistical reader. Table 3 doesn't compare like for like between unfavorable outcome sample sizes and favorable outcome sample sizes. A sample size with 10% control arm risk and 5% margin is the same whether the 10% is favorable or unfavorable outcomes. Please expand on this statement in lines 41-42 to explain how and under what circumstances this is correct.*

We disagree: a sample size with 10% control arm risk and 5% margin is not the same in the two cases: for an unfavourable outcome, the frontier point is at (10, 15), while for a favourable outcome

the frontier point is at (10, 5). We have rewritten the text to clarify that "risk" is a misnomer for a favourable outcome:

> For favourable outcomes, sample size is instead smaller when based on the risk ratio (better termed the success ratio) than on the risk difference[11].

**Minor comments:**

5. *The CONSORT guidelines recommend reporting of both absolute and relative effect sizes in a clinical trial report. If an investigator uses the SAFE frontier, would this recommendation still apply?*

Reporting both absolute and relative effect measures is very helpful in a superiority trial, but it's not so helpful in NI trials, since different effect measures will give different results (problem 1), and it only makes sense to report results on the scale of the margin. For example, STREAM only reported the risk difference. Our proposal here would similarly be to report the initially selected effect measure (usually the risk difference) even as the margin possibly adapts. We've added under "Proposal":

> Reporting should focus on the originally chosen effect measure.

6. *State somewhere that sample size calculations are done under the assumption that the expected risk in the experimental arm is the same as, and varies with, the expected risk in the control arm.*

We've done this in the very first SS calculation by adding:

> Further assuming the same true risk in both arms and no loss to follow-up …

7. *Suggest moving the OVIVA trial example to the section 'Problem 2' rather than 'Solution 2' as it might help the reader understand the problem better.*

We've gone further – we've moved it right up to "Problem 1", and linked both problems to this motivating trial.

8. *Consider adding power for SAFE frontier to Figure 1.*

We feel this would complicate the paper: we only wanted to indicate SAFE as a possible method. Instead we have referred the reader to ref 13.

9. *Suggest including a real trial example for 'Problem 1' to help the reader appreciate the problem.*

We have described OVIVA at this point. We agree this makes it easier to follow.

**Reviewer: 2**

Dr. Nan van Geloven, Leiden University Medical Center

*I appreciate the opportunity to review this manuscript on the challenge of control risk dependency in non-inferiority trials. I agree with the authors that this is an often overlooked aspect and while the methods presented in the manuscript have been published in statistical literature, it is good that authors wrote this paper targeted at a more broader readership.*

*In general I think the paper addresses the audience well, in the sense that statistical points are explained accessibly with clear numerical examples of when NI results differ between different effect measures. I do think at some points too much detail is removed. I indicate those places below. I think they need more attention.*

1. *The solution to 'problem 1' is suggested to be solved by prespecifying the scale. I think this is typically done in trial protocols, but the problem comes back in the analysis phase where eg due*

*to the desire to use a regression approach reporting changes to a different effect measure. I think this needs to be discussed too.*

We have addressed this in the discussion:

> We assumed an analysis which simply compares risks. Power may be gained by adjusting for baseline covariates. This is usually done in a logistic regression analysis, which outputs conditional odds ratios: the results should be converted to the chosen effect measure (e.g. risk difference or risk ratio) using standardisation[18].

2. *In light of this, regression approaches often lead to odds ratios. Authors do not consider this effect measure. Do they advise against it?*

We have addressed this in paragraph 1 of the discussion:

> Another effect measure for binary data is the odds ratio. This gives very similar results to the risk ratio for all examples in this paper, since the odds ratio and risk ratio perform very similarly for low-frequency outcomes[14]. In general, a non-inferiority margin expressed as an odds ratio is hard to interpret[15], and the risk difference or risk ratio should be preferred in non-inferiority trials.

3. *In problem 2: not always clear when authors consider interim analysis and when final analysis.*
    a. *Eg P1L52 'eventually', does this exclude interim?*
    b. *P3L54: 'is importantly different', does that mean at interim at final analysis or in population?*
    c. *P4L24 'after the trial has started'.*
    d. *For interim analysis, the issue of whether or not to deblind the results (needed to actually observe the control risk) is not discussed.*
    e. *And in table 4 it is advised at interim analysis to 'compare the observed control risk with that anticipated'. Without further referencing to issues with deblinding, this could be interpreted as an encouragement to deblind the data at interim analysis. I don't think that is advisable in general, can authors clarify this?*

We agree.

In response to a and c, we're being deliberately broad here, to include interim and final. We've changed a to "the observed control risk…", and we think c is now clear.

In response to b, d and e, we have revised the first paragraph of problem 2, in particular bringing out the point that the overall risk in unblinded data may often be taken as the control risk:

> Despite investigators' best efforts to anticipate the control risk correctly, sometimes its observed value differs importantly from what was anticipated. At interim analysis, this may be observed by an independent data monitoring committee with access to unblinded data or by the investigators using the pooled data and assuming no treatment effect. At final analysis, it will be clear to all.

4. *Relatedly, there is little attention in the paper to type I error control. Probably specific procedures needed for this when using the SAFE frontier? Can this be added to one of the examples or the figure 2? End of 'limitations' section seems to suggest no good method exists for it yet? Can it then be used already in practice, what is the current advise on it? I also wonder if the advice to seek funding to enlarge the trial based on observed control risk during interim (table 4) will have any consequences for type I error control. And the same worry for the advice to bring the final analysis forward in case of power advantage anticipated at interim analysis.*

We agree that we gave the impression that T1ER control is an unsolved problem. We've now explained this better: we know how to control T1ER with margin re-estimation, but not with sample size re-estimation or earlier analysis. Details are beyond the scope of this paper. The discussion now reads:

> We described a non-inferiority margin modification procedure based on the SAFE frontier and overall blinded data. Non-inferiority margin modification alone can lead to type 1 error inflation, but special analysis methods can control the type 1 error rate[13]. Sample size re-estimation or bringing final analysis time forward could have unexplored implications for type 1 error.

5. *In the suggested wording in the Box, there is no mentioning of the actual margin, neither of the location of points B. This seems prone to incomplete reporting if researchers would copy this text.*

We have corrected this.

6. *The end of the paper is very short and only mentions something about the SAFE frontier. Intro of the paper seems to address a more broad problem, not only the SAFE frontier. The closing of the paper could use some more attention and should link to intro.*

We have expanded the end of the paper substantially, with longer discussion and a conclusion.

7. *The example code in the appendix should include the full write out of the default arguments, eg alpha, one or 2 sided, positive or negative event, etc.*

We have added the following explanation:

> # The commands below assume a 2.5% one-sided significance level, 90% power and 1:1 allocation: these can be changed by the sig.level=, power= and r= options
>
> # They also assume a favourable outcome. For an unfavourable outcome we would specify the unfavourable=FALSE option and set the NI margin to be negative

**Some further minor suggestions for the paper:**

8. *in some places the issue is described as 'the control risk problem', eg title and P6L1. While in the paper '2 problems' are mentioned. This wording is bit confusing. See also P4L24 'to avoid these problems'. Both problem 1 and problem 2?*

We agree. We now always refer to two problems, including in the title and standfirst.

9. *P2L9 'broadening therapeutic toolkit': vague wording. Also the setting where non-inferiority is used to indirectly prove superiority over placebo is not mentioned/referred to.*

We've dropped the vague wording. The second setting is now handled in the introduction:

> A different objective of non-inferiority trials, important in a regulatory context but not considered here, is to demonstrate that a new intervention gives better disease outcomes than placebo without directly comparing it with placebo[2].

10. *P4L6: 'are only the same'. They are never the same, maybe better to reword into 'will both be true at the same time' or smth like that?*

Thank you, we've changed this to

> The discrepancy arises because a risk difference of 5% and a risk ratio of 2 are only simultaneously true if the control risk is exactly 5%.

11. *P5L36: should determine upfront*

We agree but we've used "at trial design stage".

12. *P5L50: may not be clear to all readers what 'this procedure' means, it is worthwhile to spell out*

Good point, we've changed to "Analysis using a possibly modified margin".

13. *P5L45: it was not clear from the example so far that risk difference was the originally preferred scale. Also not so clear where to place points B, and why plus or minus 2%?*

First point, done. Second point, we've clarified that this is just an example of a SAFE frontier, and added:

> Choice of points A and B should reflect clinical understanding, but placing points B close to the frontier point inflates sample size.

14. *P5L48: 'typically, we expect one of the frontiers in the Figure would be adequate.' Why is this, there can be many others? I think the point is more that trialists need to be aware that they need to choose a curve, not only a point. They can in principle choose any, but these are 3 simple candidates?*

We've dropped this sentence.

15. *table 4 design: here referred to 'scale' at other places 'effect measure'. I suggest to unify. Also here needs to be clear that the size of NI margin needs to be specified.*

Changed to "Choose an effect measure and non-inferiority margin which are clinically reasonable and statistically efficient."

16. *in table 4 indicated that it is uncommon that different control risk leads to higher power. Why would than be, is it not a symmetric problem?*

We have removed this wording.

17. *Figure 1: legend needs to be clear about that risk ratio is for negative or positive outcome*

Thank you, we've added "with unfavourable outcome" to the caption.

**Reviewer: 3**

Prof. Richard Riley, University of Birmingham

*This is a well-written and important article that I think would be well cited and have an immediate impact. It would fit well as a methodology piece in the journal, as introduces interesting issues/problems with sample size and non-inferiority trials, with potential solutions that are well justified. I see that two reviewers already given pertinent comments, so I only have a few suggestions to add*

1. *Add a concluding remarks section, just to summarise the overall article and what has been discussed – otherwise ends rather abruptly*

Done.

2. *The word estimand is not used, which I am surprised about. I think the issues should be framed in terms of the estimand for the NI trial, and how this might need to change depending on the particular control risk issue that arises*

We had avoided use of the E-word in order to be more clinician-friendly, but we agree it is an important and helpful idea. Problem 1 requires choice of estimand and Problem 2 requires modification of margin without change of estimand. We've therefore added in Solution 1 after "We recommend stating the intended effect measure explicitly.":

> This is part of defining the estimand, alongside stating the population, treatments being compared, outcome measure (including timepoint) and handling of intercurrent events[10].

3. *Odds ratios are not considered, but many trials focus on the OR as an estimand. How does this impact things?*

We have addressed this in paragraph 1 of the discussion, saying that the odds ratio behaves very much like the risk ratio in the examples of this paper, and is harder to interpret otherwise:

> Another effect measure for binary data is the odds ratio. This gives very similar results to the risk ratio for all examples in this paper, since the odds ratio and risk ratio perform very similarly for low-frequency outcomes[14]. In general, a non-inferiority margin expressed as an odds ratio is hard to interpret[15], and the risk difference or risk ratio should be preferred in non-inferiority trials.

4. *Similarly, if the estimand is a conditional effect (e.g. adjusted for baseline age, stage of disease), how might this impact the issues and solutions?*

As noted above, we now recommend against using the odds ratio scale for NI trials, and in favour of the RD and RR. Since RD and RR are collapsible estimands, adjustment is an estimation issue and does not change the estimand. We have added a paragraph in the discussion to stress that use of logistic regression does not force reporting on the odds ratio scale:

> We assumed an analysis which simply compares risks. Power may be gained by adjusting for baseline covariates. This is usually done in a logistic regression analysis, which outputs conditional odds ratios: the results should be converted to the chosen effect measure (e.g. risk difference or risk ratio) using standardisation[18].

5. *Could we have a box of recommendations perhaps?*

This is table 4.

6. *Worth emphasising that risk by a particular time-point, for both binary or tte outcomes, and so the estimand needs to be defined in terms of risk by timepoint stated?*

Added in the estimand sentence (see point 2 above).

7. *The title mentions the word 'resilience', but this is not referred to in the actual paper. It's not clear what is meant by this – so perhaps 'Tackling the control risk in non-inferiority trials' would be better, but this is a minor point (tackle is mentioned 4 times!)*

We have changed the title to "Tackling control risk problems in non-inferiority trials".
